# Factores asociados a los reclamos en una aseguradora de servicios de salud en Colombia

**DOI:** 10.1016/j.aprim.2025.103378

**Published:** 2026-02-09

**Authors:** Betsy Sánchez-Aponte, Diana Vargas-Gamboa, Alba Páez-Páez, Diana Sánchez-Calderón

**Affiliations:** Facultad de Medicina, Universidad El Bosque, Bogotá, Colombia

La Ley Estatutaria en Colombia que reguló el derecho fundamental a la salud estableció mecanismos de acceso a los servicios con oportunidad, eficacia y calidad, donde los usuarios pueden presentar peticiones, quejas, reclamos y solicitudes (PQRS), en caso de considerar vulnerado su derecho, ante las entidades prestadoras de salud (EPS) o entes de inspección, vigilancia y control^1^, ^2^. La Superintendencia Nacional de Salud reportó que los principales motivos que generaron reclamos en salud durante el año 2023 en Colombia estuvieron relacionados con barreras de acceso a servicios de salud (84,9%) y a tecnologías en salud (90%)^3^.

El objetivo de este estudio fue analizar los condicionantes de peticiones, reclamos y solicitudes (PQRS) en una Entidad Administradora de planes de beneficios en Salud (EAPB) en Colombia durante el año 2023.

El diseño empleado para este estudio fue de tipo observacional retrospectivo

Se analizaron 47.992 registros de peticiones, quejas, reclamos y solicitudes del año 2023. Se excluyeron los registros con información incompleta que no permitieran identificar la causa, el servicio que originó la PQRS y el tipo de reclamo.

Para el análisis de datos se utilizó el programa estadístico SPSS 29.0®. Se generaron medidas de frecuencia, tendencia central y dispersión de acuerdo con el tipo de variable. Se realizó un análisis bivariado. Se utilizó la prueba chi-cuadrado para las variables de desenlace nominal y la prueba de Fisher para las variables de desenlace polinomial. Aquellas variables evaluadas que tuvieron un valor de p menor o igual a 0,2 se incluyeron en un modelo de regresión logística polinomial para determinar si se trataban de variables confusoras o si se consideraban variables asociadas al desenlace de forma significativa. Se consideró un valor de p estadísticamente significativo menor a 0,05.

Las principales causas de las PQRS estuvieron relacionadas con oportunidad de atención en medicina especializada (79,7%) y acceso a medicamentos (11,9%) (tabla 1).

**Tabla 1:** Causas y fuentes de origen de las PQRS

Causa	Fuente	n = 47.992	%
Oportunidad de atención	Consulta especializada	22.915	59,9
	Apoyo diagnóstico	8.224	21,5
	Procedimientos quirúrgicos	2.543	6,6
	Servicios de apoyo	1.938	5,1
	Atención hospitalaria	1.369	3,6
	Especialidades pediatría	577	1,5
	Trámites administrativos	498	1,3
	Consulta básica	126	0,3
	No definida	57	0,1
	Medicamentos	13	0
	Tratamiento alto costo	11	0
	Agresiones	1	0
Acceso a medicamentos	Medicamentos	5.362	93,9
	Servicios de apoyo	236	4,1
	Tratamiento alto costo	107	1,9
	Consulta especializada	5	0,1
	Apoyo diagnóstico	1	0
	Atención hospitalaria	1	0
	Trámites administrativos	1	0
Trámites administrativos	Trámites administrativos	2.688	99,5
	No definida	11	0,4
	Corrupción	2	0,1
	Atención hospitalaria	1	0
Causa no definida	No definida	907	100
Tutelas	Servicios de apoyo	180	61,6
	No definida	90	30,8
	Procedimientos quirúrgicos	11	3,8
	Medicamentos	11	3,8
Atención deshumanizada	Agresiones	64	100
Eventos adversos	Atención hospitalaria	22	52,4
	Consulta especializada	11	26,2
	Servicios de apoyo	7	16,7
	Procedimientos quirúrgicos	2	4,8

Fuente: Base de datos institucional. Adaptación realizada por las autoras.

Se realizó un modelo de regresión logística polinomial, donde se incluyeron las variables: red de atención de servicios de salud, grupo de priorización, tipo de requerimiento, causa de la queja, mes de radicación y grupo etario. Como desenlace principal se definió el estado de resolución de la PQRS «fuera de tiempo». Pertenecer a un grupo de priorización (mayor de 60 años), ser de sexo femenino y pertenecer a la red de prestación de servicios del norte de la ciudad favorece que la PQRS sea respondida a tiempo. La coherencia del modelo es aceptable (0,317), reconociendo que las variables finales pueden tener múltiples desenlaces.

## Conclusiones

Los puntos críticos que impactan la incidencia de PQRS estuvieron relacionados principalmente con oportunidad de atención a medicina especializada y acceso a medicamentos; estos aspectos resultan relevantes más aún en población adulta mayor, quienes en el estudio representaron el grupo con mayor radicación de PQRS y que por su ciclo de vida demandan mayor cantidad de servicios de salud.

Se encontraron algunas falencias en los registros de la base de datos, lo que impactó de manera directa el proceso de tipificación de las PQRS, generando deficiencia en la calidad de los datos.

## Financiación

Esta investigación no recibió ninguna subvención específica de agencias de financiación de los sectores público, comercial o sin fines de lucro.

## Consideraciones éticas

De acuerdo con la resolución No. 8430 de 1993 del Ministerio de Salud, por medio de la cual se establecen las normas científicas, técnicas y administrativas para la investigación en salud, el presente estudio se clasificó como una investigación de bajo riesgo. Se trata de una investigación basada en la revisión de una base de datos anonimizada de la EAPB, obtenida con previa autorización de la gerencia de la organización con un objetivo académico sin ningún tipo de intervención en seres vivos.

## Conflicto de intereses

Los autores declaran no tener ningún conflicto de intereses.

## References

[1] Ministerio de Salud y Protección Social. Ley Estatutaria 1751. 2015.

[2] Ministerio de Salud y Protección Social. Ley 1438 de 2011. 2011.

[3] Superintendencia Nacional de Salud. Comportamiento de reclamos en salud y solicitudes de información. Superintendencia Nacional de Salud; 2023.

